# A new risk calculation model for complications of hepatectomy in adults over 75

**DOI:** 10.1186/s13741-024-00366-y

**Published:** 2024-02-26

**Authors:** Lining Xu, Weiyu Wang, Yingying Xu

**Affiliations:** 1 Department of General Surgery, The Second Medical Center & National Clinical Research Center for Geriatric Diseases, Chinese PLA General Hospital, Beijing, 100853 China; 2 Zhongnan Hospital of Wuhan University, Institute of Hepatobiliary Diseases of Wuhan University, Transplant Center of Wuhan University, National Quality Control Center for Donated Organ Procurement, Hubei Key Laboratory of Medical Technology On Transplantation, Wuhan, 430071 China; 3 Department of Internal Medicine, Henan Cancer Hospital, Zhengzhou, 450003 China

**Keywords:** Hepatectomy, Risk assessment tool, Older persons

## Abstract

**Background:**

Owing to poor organ function reserve, older adults have a high risk of postoperative complications. However, there is no well-established system for assessing the risk of complications after hepatectomy in older adults.

**Methods:**

This study aimed to design a risk assessment tool to predict the risk of complications after hepatectomy in adults older than 75 years. A total of 326 patients were identified. A logistic regression equation was used to create the Risk Assessment System of Hepatectomy in Adults (RASHA) for the prediction of complications (Clavien‒Dindo classification ≥ II).

**Results:**

Multivariate correlation analysis revealed that comorbidity (≥ 5 kinds of disease or < 5 kinds of disease, odds ratio [OR] = 5.552, *P* < 0.001), fatigue (yes or no, OR = 4.630, *P* = 0.009), Child‒Pugh (B or A, OR = 4.211, *P* = 0.004), number of liver segments to be removed (≥ 3 or ≤ 2, OR = 4.101, *P* = 0.001), and adjacent organ resection (yes or no, OR = 1.523, *P* = 0.010) were independent risk factors for postoperative complications after hepatectomy in older persons (aged ≥ 75 years). A binomial logistic regression model was established to evaluate the RASHA score (including the RASHA scale and RASHA formula). The area under the curve (AUC) for the RASHA scale was 0.916, and the cut-off value was 12.5. The AUC for the RASHA formula was 0.801, and the cut-off value was 0.2106.

**Conclusion:**

RASHA can be used to effectively predict the postoperative complications of hepatectomy through perioperative variables in adults older than 75 years.

**Trial registration:**

The Research Registry: researchregistry8531. https://www.researchregistry.com/browse-the-registry#home/registrationdetails/63901824ae49230021a5a0cf/.

## Introduction

Although young and healthy patients may be able to withstand surgical trauma and recover quickly after surgery, older persons may suffer from significant challenges (Myers and Fonda 2016). Despite advances in surgical and anesthetic techniques and perioperative management, postoperative complications in older persons remain an important cause of increased mortality and medical costs. Older persons have low immunity, many perioperative comorbidities, degenerative changes in important organ functions, and reduced reserve and compensation capacities. These factors can lead to much greater postoperative complication risks in older persons than in young persons. Perioperative management is crucial for ensuring the success of surgery in older persons. Geriatric surgery has specific considerations, which are reflected in various aspects before, during, and after surgery. With the participation of multiple disciplines, a careful understanding of the patient’s condition and a comprehensive assessment of older persons' tolerance to surgery play important roles in the postoperative safety of older patients (González-Montalvo et al. 2020).

Older age is an independent risk factor for complications during liver surgery (Menon et al. 2006). A study analyzed the clinical data of 2397 patients who underwent hepatectomy. The 90-day mortality rate, 30-day mortality rate, and myocardial infarction rate in patients aged ≥ 80 years were 13.3%, 5.6%, and 7.9%, respectively. Multivariate analysis revealed that age ≥ 80 years was significantly associated with 90-day mortality (Mueller et al. 2021). A study of 7621 patients showed that elderly patients (aged ≥ 75 years) experienced higher rates of severe complications (23.9% versus 18.4%; *P* < 0.001) and overall postoperative mortality (4.8% versus 2.0%; *P* < 0.001). The occurrence of any severe complication was associated with a mortality rate of 20.1% in elderly patients and 10.8% in nonelderly patients (*P* < 0.001) (Tzeng et al. 2014). Age (65 years and older) is also an independent risk factor for the loss of self-care ability among older patients after liver surgery (Lallement et al. 2020).

In summary, preoperative evaluation of liver surgery in older persons is important (Xu et al. 2017). With the aging of the world’s population, more elderly people need surgical treatment. However, due to the many comorbidities in elderly individuals, there are many complications, such as organ dysfunction after surgery, and the mortality rate is also higher in these patients than in young individuals, which seriously affects the prognosis and quality of life of older persons. Therefore, it is necessary to explore methods for predicting the postoperative outcome of older persons before surgery. For high-risk older persons, timely intervention should be given before surgery to improve the factors affecting postoperative adverse events and reduce the incidence of postoperative complications and mortality. However, there is currently no perfect evaluation system for the risk of liver surgery in older persons. Therefore, establishing a risk evaluation system for postoperative complications and improving the safety of hepatectomy in older persons has become an urgent clinical problem.

This study aimed to design a risk assessment system to predict the risk of complications after hepatectomy in adults older than 75 years. Furthermore, the cut-off values for the incidence of complications were summarized to facilitate more accurate and intuitive preoperative evaluations.

## Methods

### Study design

This is a case–control study of patients (aged ≥ 75 years) undergoing hepatectomy for whom complete medical records were available at 3 district general hospitals across China between January 2013 and December 2022. All the institutions obtained their respective approval according to their local hospital’s requirements. This study was approved by the Ethics Committee of Chinese PLA General Hospital (S2022-664–01). All procedures performed in studies involving human participants were in accordance with the ethical standards of the Ethics Committee of Chinese PLA General Hospital and with the 1975 Helsinki Declaration and its later amendments or comparable ethical standards, and the need for informed consent was waived by the Ethics Committee of Chinese PLA General Hospital. This retrospective study did not contain any identification information about the patients, so informed consent was not needed.

### Setting and participants

A total of 326 patients were identified between January 2013 and December 2022 at 3 district general hospitals (located in Beijing, Zhengzhou, and Wuhan, China). One hundred patients were randomly selected to constitute the validation cohort, and the other patients were randomly assigned to the derivation cohort. There were 226 patients in the derivation cohort. The patient backgrounds of the individuals in the derivation cohort and the validation cohort are shown in Table 1.

**Table 1:** The patient backgrounds of the derivation cohort and the validation cohort

Factors	Derivation cohort	Validation cohort
General background		
Age (years)	78.3 ± 2.69	77.7 ± 2.28
Gender		
Female	70 (31.97%)	36 (36.00%)
Male	156 (69.03%)	64 (64.00%)
Comorbidity		
< 5 kinds of diseases	149(65.93%)	70(70.00%)
≥ 5 kinds of diseases	77(34.07%)	30(30.00%)
History of dementia		
No	204(90.27%)	89(89.00%)
Yes	22(9.73%)	11(11.00%)
History of anxiety/depression		
No	210(92.92%)	94(94.00%)
Yes	16(7.08%)	6(6.00%)
Weight loss		
No	179(79.20%)	81(81.00%)
Yes	47(20.80%)	19(19.00%)
Fatigue		
No	65(28.76%)	27(27.00%)
Yes	161(71.24%)	73(73.00%)
Diagnosis		
Malignant diseases	183(80.97%)	83(83.00%)
Benign diseases	43(19.03%)	17(17.00%)
Reoperation		
No	220(97.35%)	97(97.00%)
Yes	6(2.65%)	3(3.00%)
Blood test		
Albumin (g/L)	38.18 ± 4.26	38.63 ± 4.74
Alpha-fetoprotein (ng/ml)	723.44 ± 3160.87	445.61 ± 2514.44
Total bilirubin (μmol/L)	17.65 ± 30.16	16.34 ± 22.30
Alkaline phosphatase (U/L)	175.88 ± 208.13	154.77 ± 175.12
Hepatitis B surface antigen		
Negative	156(69.03%)	65(65.00%)
Positive	70(30.97%)	35(35.00%)
Hepatitis C surface antigen		
Negative	212(93.81%)	95(95.00%)
Positive	14(6.19%)	5(5.00%)
Cirrhosis		
No	183(80.97%)	78(78.00%)
Yes	43(19.03%)	22(22.00%)
Child–pugh		
A	191(84.51%)	87(87.00%)
B	35(15.49%)	13(13.00%)
BMI(kg/m^2^)		
< 18.5	44(19.47%)	23(23.00%)
≥ 18.5	182(80.53%)	77(77.00%)
Operation plan		
Adjacent organ resection		
No	218(96.46%)	95(95.00%)
Yes	8(3.54%)	5(5.00%)
Number of segments resected		
≤ 2	147(65.04%)	66(66.00%)
≥ 3	79(34.96%)	34(34.00%)
Resection style		
Nonanatomical	107(47.35%)	54(54.00%)
Anatomical	119(52.65%)	46(46.00%)
Operative duration (min)		
< 180	119(52.65%)	53(53.00%)
≥ 180	107(47.35%)	47(47.00%)
Blood loss (mL)		
≤ 800	210(92.92%)	91(91.00%)
> 800	16(7.08%)	9(9.00%)

### Definitions

All study participants had clear surgical indications, and the first choice of treatment was surgery. Therefore, no neoadjuvant treatment was administered before the procedure. For specific surgical indications for malignant liver tumors, the “Chinese guidelines for the diagnosis and treatment of primary liver cancer” were referred to Zhou, et al. (2017). The benign lesions in this study mainly included hepatolithiasis and hepatic haemangiomas. In China, hepatolithiasis is the most common benign liver disease that requires surgery. Patients with hepatolithiasis often develop infection and abnormal liver function because of their special pathological characteristics. These complications can lead to local liver damage, and partial hepatectomy is required in these patients. Patients with other benign conditions, including hepatic haemangiomas, require surgery because of the large volume of the lesion, which compresses the surrounding organs and causes abdominal distension and other clinical symptoms.

Liver dysfunction after hepatectomy is an important cause of perioperative death. In this study, the “individualized evaluation and decision-making system for the safety limit of hepatectomy” proposed in the Chinese “consensus on evaluation of hepatic functional reserve before hepatectomy” was used to evaluate liver reserve function (Dong, et al. 2011). Only those patients who met the surgical indications underwent surgery.

### Selection of the input variables

Detailed medical records were available for all included patients. The preoperative variables we focused on in this study included basic demographic data, diagnosis, laboratory examination, medical history, and the state of frailty. The main variables included sex and Child‒Pugh grade; all of these variables were included as candidate variables in this study (Hamaoka et al. 2017). The planned intraoperative factors included the excision scope and surgical procedure. This study did not include variables related to specific surgical procedures, such as prolonged hepatic pedicle occlusion. The main reason is that with the advancement of surgical techniques, surgeons are currently able to complete surgical procedures within a very short hepatic pedicle occlusion time without causing damage to liver function (Yoshino et al. 2021). Further details are provided in Table 1.

Comorbidities and fatigue are the main conditions used to assess frailty. In this study, patients were diagnosed with more than five kinds of diseases [hypertension, diabetes, cancer (other than minor skin cancer), chronic lung disease, heart attack, chronic heart failure, angina, asthma, arthritis, stroke, and kidney disease.] and fatigue (refers to fatigue for most of the previous 4 weeks) were used as candidate indicators of risk factors according to the FRAIL scale (Church et al. 2020; Thompson et al. 2020).

### Selection of the output/outcome variable

Complications refer to the occurrence of another disease or symptom caused by a disease during the process of disease development; the latter is a complication of the former. Postoperative complications were considered those occurring within 30 days after surgery. Postoperative complications were defined as complications after surgery with a Clavien‒Dindo classification (Clavien et al. 1992; Clavien et al. 2009; Clavien et al. 1992; Dindo et al. 2004) of surgical complications ≥ II.

### Statistical analysis

The Risk Assessment System for Hepatectomy in Adults (RASHA) includes two parts: the scale of postoperative complication prediction scale (RASHA scale) and the calculation formula for postoperative complication probability (RASHA formula).

### Establishment of the RASHA scale

Postoperative complications (Clavien‒Dindo grade ≥ II) were defined as positive results. The independent variables were the aforementioned risk factors to be screened. The variable grades were established according to the values of categorical variables such as age, serum ALB concentration, and bilirubin concentration. All factors were included in the multivariate logistic regression analysis. The risk index (odds ratio, OR) was assigned to the nearest whole number according to the principle of rounding. The sum of the risk scores of all risk factors for a single patient was defined as the total risk score for the patient's complications. The risk indices of all patients with complications were calculated to establish the complication risk assessment scale (RASHA scale).

### Establishment of the RASHA formula

A logistic regression equation was used to design the RASHA formula for the prediction of postoperative complications: *P* = 1/{1 + exp[-(α + β_1_χ_1_ + β_2_χ_2_ + …..β_n_χ_n_)]}, where *P* represents the probability of complications; thus, when *P* = 1, the probability of complications was 100%. α is a constant term, β_1_–β_n_ is the regression coefficient corresponding to the risk index of complication risk factors, and χ_1_–χ_n_ is the risk index grade of the complication risk factors.

### Methods to verify RASHA

RASHA was used to score and calculate the probability of postoperative complications. The receiver operating characteristic (ROC) curve was used to evaluate the resolution of RASHA, the area under the curve (AUC) was calculated, and the cut-off value of the risk index was calculated.

The statistical software SPSS, version 25.0 (IBM Corp., Armonk, NY, USA), was used for the data analysis. Continuous variables are expressed as the mean ± SD. Categorical variables were compared using the chi-squared test. Univariate analysis ANOVA was used to analyze the relationship between the complications of patients who underwent hepatectomy and perioperative factors. Multivariate analysis was performed on the factors related to the complications of patients who underwent hepatectomy via logistic regression. *P* < 0.05 was considered to indicate statistical significance.

## Results

In the derivation cohort of 226 patients, 49 (21.68%) developed complications (Clavien‒Dindo ≥ II), 10 (20.41%) of whom had more than two kinds of complications. Five fatal complications occurred, accounting for 10.20% of the total complications, for a mortality rate of 2.21%. The results of the univariate analysis related to complications are shown in Table 2. Further details of the complications are provided in Table 3.

**Table 2:** The patient characteristics and univariate analysis of the perioperative factors associated with hepatectomy complications

Factors	*Complicated*	*Uncomplicated*	*P *value
General background			
Age (years)	79.4 ± 3.36	78.0 ± 2.40	0.002
Gender			
Female	5	65	< 0.001
Male	44	112	
Comorbidity			
< 5 kinds of diseases	6	143	0.001
≥ 5 kinds of diseases	43	34	
History of dementia			
No	40	164	0.026
Yes	9	13	
History of anxiety/depression			
No	43	167	0.120
Yes	6	10	
Weight loss			
No	29	150	< 0.001
Yes	20	27	
Fatigue			
No	8	57	0.034
Yes	41	120	
Diagnosis			
Malignant diseases	41	142	0.587
Benign diseases	8	35	
Reoperation			
No	49	171	0.999
Yes	0	6	
Blood test			
Albumin (g/L)	36.33 ± 4.40	38.69 ± 4.09	0.001
Alpha-fetoprotein (ng/ml)	1558.21 ± 5121.70	542.78 ± 2542.10	0.117
Total bilirubin (μmol/L)	33.27 ± 52.13	13.33 ± 18.31	< 0.001
Alkaline phosphatase (U/L)	226.15 ± 205.84	164.75 ± 207.68	0.126
Hepatitis B surface antigen			
Negative	39	117	0.080
Positive	10	60	
Hepatitis C surface antigen			
Negative	47	165	0.396
Positive	2	12	
Cirrhosis			
No	32	151	0.002
Yes	17	26	
Child–Pugh			
A	35	156	0.006
B	14	21	
BMI (kg/m^2^)			
< 18.5	20	24	< 0.001
≥ 18.5	29	153	
Operation plan			
Adjacent organ resection			
No	41	177	0.003
Yes	8	0	
Number of segments resected			
≤ 2	20	127	< 0.001
≥ 3	29	50	
Resection style			
Nonanatomical	24	83	0.796
Anatomical	25	94	
Operative duration (min)			
< 180	10	99	< 0.001
≥ 180	39	78	
Blood loss (mL)			
≤ 800	42	168	< 0.001
> 800	7	9

**Table 3:** Postoperative complications

Complication	*n*	Clavien-Dindo classification
Infection in the abdomen		
Around drainage tube	1	II
Intra-abdominal abscess	2	III
Peri-liver abscess	3	III
Bile duct		
Biliary tract obstruction	1	III
Bile leakage	3	III
Bleeding		
Incision bleeding	1	II
Alimentary tract hemorrhage	1	II
Abdominal cavity/raw surface bleeding	4	III or V
Surgical site-related injuries		
Incision disruption	1	III
Liver and kidney inadequacy		
Hepatic inadequacy	8	IV
Renal inadequacy	2	IV
Pulmonary and cardiovascular		
Heart failure	1	II
Heart infarction	3	II or V
Respiratory tract infection	9	II
Deep venous thrombosis (lower extremity)	2	II
Respiratory insufficiency	3	IV
Atelectasis	2	III
Pneumonia	9	II
Pleural effussion	7	II or III
Others		
Cerebral accident	1	II
Ventricular fibrillation	1	V
Stress ulcer	1	II

### RASHA scale

Multivariate correlation analysis revealed that the independent influencing factors of postoperative complications of hepatectomy in older persons (aged ≥ 75 years) were comorbidities (≥ 5 kinds of disease or < 5 kinds of disease, OR = 5.552, *P* < 0.001), fatigue (yes or no, OR = 4.630, *P* = 0.009), Child–Pugh (B or A, OR = 4.211, *P* = 0.004), number of liver segments to be removed (≥ 3 or ≤ 2, OR = 4.101, *P* = 0.001), and adjacent organ resection (yes or no, OR = 1.523, *P* = 0.010). A scoring scale was used for these factors, which were rounded to be clinically useful as follows: comorbidity ≥ 5 kinds of disease = 6 points, fatigue = 5 points, Child‒Pugh B/C = 4 points, number of liver segments to be removed ≥ 3 = 4 points, and adjacent organ resection = 2 points, as shown in Table 4.

**Table 4:** Multivariate analysis of the perioperative factors associated with the hepatectomy complications and new model

Variable	Intercept	Wald	Odds ratio	*P* value	Condition	Score
χ_1_: Comorbidity	1.714(β_1_)	12.241	5.552	< 0.001	< 5 kinds of disease ≥ 5 kinds of disease	1 6
χ_2_: Fatigue	1.531(β_2_)	6.765	4.630	0.009	No Yes	1 5
χ_3_: Child–pugh	1.438(β_3_)	8.406	4.211	0.004	A B	1 4
χ_4_: Number of segments resected	1.411(β_4_)	10.812	4.101	0.001	≤ 2 ≥ 3	1 4
χ_5_: Adjacent organ resection	0.421(β_5_)	6.669	1.523	0.010	No	1
					Yes	2
Constant	− 3.927(α)	11.765				

### RASHA formula

Using the preoperative risk factor score for each hepatectomy complication as the independent variable and positivity/negativity for complications as the dependent variable, a binomial logistic regression model was established. The specific intercepts (regression coefficient values) are shown in Table 4. The calculation formula for complication risk is as follows:

$$P=\;1/\;\left\{1+\exp\left[-\left(-3.927\;+{\;1.714}{\chi_{\mathit1}}\;+\;{1.531}{\chi_{\mathit2}}\;+\;{1.438}{\chi_{\mathit3}}\;+\;{1.411}{{\mathrm\chi}_4}\;+\;{0.421}{\chi_{\mathit5}}\;\right)\right]\right\}$$

**Fig. 1:**
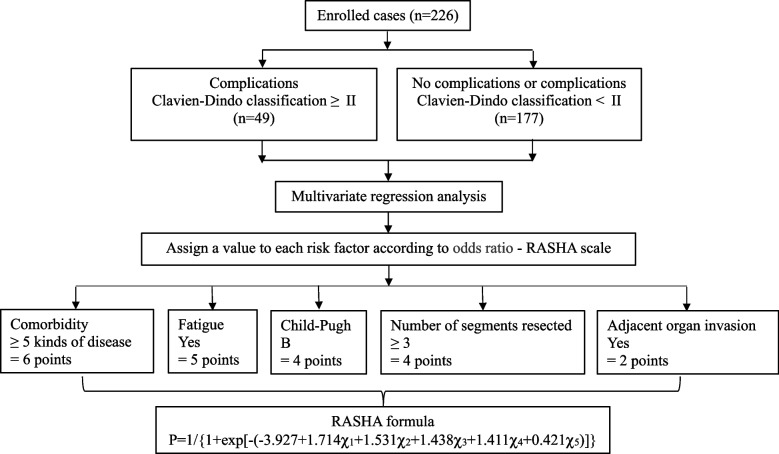
Flow diagram

A flow diagram of this study is shown in Fig. 1.

### Predictive efficacy of the RASHA scale

**Fig. 2:**
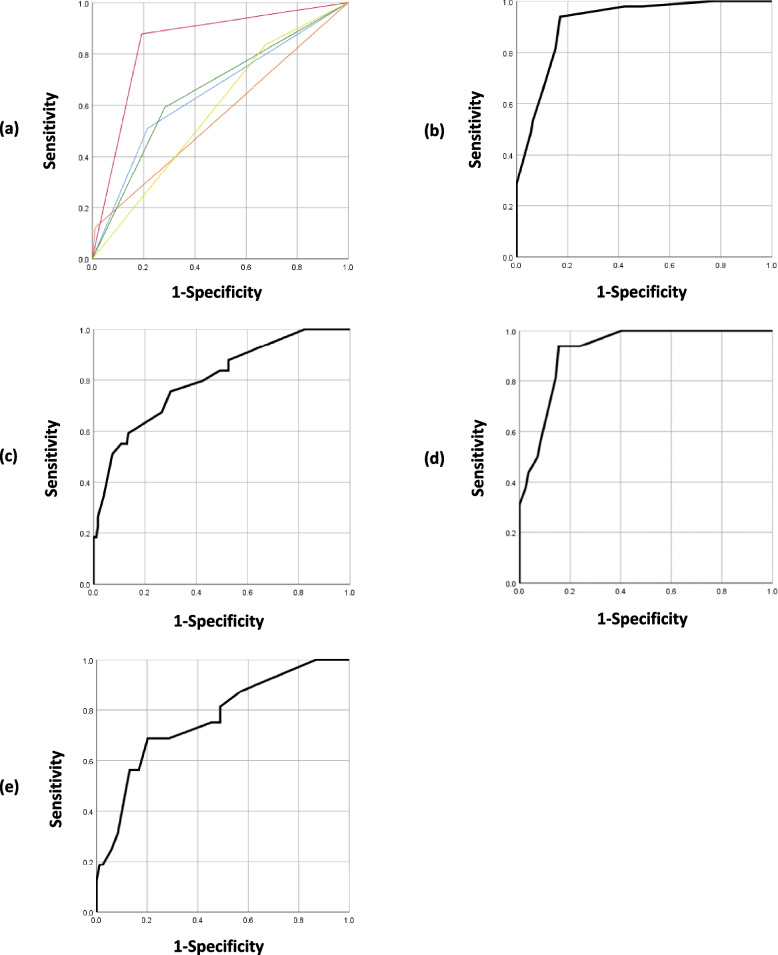
Predictive efficacy of the RASHA scale/formula. **a** Predictive efficacy of the five variables. Blue line: Child–Pugh; Purple line: comorbidity; green line: number of segments resected; red line: adjacent organ resection; yellow line: fatigue. Predictive efficacy of the five variables: The area under the curve (AUC) were 0.648, 0.843, 0.655, 0.556, and 0.579 for the variables: Child–Pugh, comorbidity, number of segments resected, adjacent organ resection, fatigue, respectively. **b** Predictive efficacy of the RASHA scale. The AUC of the RASHA scale was 0.916. **c** Predictive efficacy of the RASHA formula. The AUC of the RASHA formula was 0.801. **d** Validation of the RASHA scale. The AUC of the RASHA scale was 0.922. **e** Validation of the RASHA formula. The AUC of the RASHA formula was 0.766

The ROC curves for the identified independent risk factors are plotted in Fig. 2a. The ROC curve of the five combined variables (RASHA scale) is shown in Fig. 2b. The AUC of the RASHA scale was 0.916, and the corresponding standard error was 0.020. The cut-off value of the total score, calculated by adding the values of all risk factors, was 12.5. With this threshold, the incidence of postoperative complications was significantly different between the groups with ≤ 12 points and those with ≥ 13 points (*χ*2 = 101.753, *P* < 0.001).

### Predictive efficacy of the RASHA formula

The scores of the independent risk factors for each patient were substituted into the RASHA formula to calculate the expected probability of complications, and the ROC curve was used to evaluate predictive efficacy, as shown in Fig. 2c. The AUC of the RASHA formula was 0.801, and the corresponding standard error was 0.036. The cut-off value for the expected probability of complications was 0.2106.

### Validation of RASHA

A total of 100 patients were enrolled in the validation cohort to verify the validity of the RASHA scale. The discrimination ability of the nomograms was analyzed using ROC curves. The AUC of the RASHA scale was 0.922 (Fig. 2d). The AUC for the RASHA formula was 0.766 (Fig. 2e).

## Discussion

Several studies have shown that age is a relevant factor for complications in liver surgery (Trundle et al. 2019; Laporte and Kalil 2013). With increasing age, the complication rate of liver surgery has gradually increased (Liu et al. 2021). A study of 663 patients who underwent hepatectomy showed that the 90-day mortality rates were 11.0%, 13.0%, and 17% for patients aged > 70, 75, and 80 years, respectively, and that the complication rates were 53%, 57%, and 66%, respectively (Shutt et al. 2016). Therefore, establishing a liver surgery complication evaluation system for older persons and dealing with the risk factors for complications are important.

Although advances in surgical techniques and perioperative management have reduced the incidence of complications and the mortality rate after hepatectomy during the last half century, liver failure after major hepatectomy has remained an important problem (Ocak et al. 2020). Before the 1980s, the mortality rate related to hepatectomy was approximately 10%. In recent years, however, the mortality rate has decreased to < 1% in some surgical centers, and several recent studies have reported a mortality rate of 0 (Huang et al. 2009). The incidence of liver failure after hepatectomy varies greatly in the reported literature, and the generally accepted incidence is between 8 and 12% (Søreide and Deshpande 2021). Liver failure after hepatectomy is the most common cause of death after liver surgery, and a decrease in liver function in older persons before surgery may be one of the reasons for this situation (Lodewick et al. 2017). Therefore, a detailed preoperative assessment of liver function is important. The Child–Pugh grade is the most widely used indicator of liver function (Huang and Gao 2020). This study showed that Child‒Pugh grade B was an independent risk factor for postoperative complications in older persons (aged ≥ 75 years).

The residual liver volume after hepatectomy is a key predictor of perioperative outcomes (Simpson et al. 2014). It is closely related to various barriers, including postoperative ascites, bleeding, and wound healing (Blüthner et al. 2020). The residual liver volume can be used to predict the risk of liver failure in patients undergoing hepatectomy (Olthof et al. 2019). Although the etiology of liver failure after hepatectomy is multifactorial, insufficient residual liver volume is considered to be the most important modifiable predictor. Preoperative assessment of residual liver function and volume is essential before liver resection (Khan et al. 2018). Unless the remaining liver after hepatectomy has a sufficient volume, surgery may lead to liver dysfunction, which may, in turn, lead to further postoperative complications. With increasing age, liver volume and blood flow are significantly reduced. In addition, the liver reserve function of older patients is significantly decreased, which reduces their tolerance to liver disease treatment (Tajiri and Shimizu 2013). This study showed that ≥ 3 liver segments removed was an independent risk factor for complications after hepatectomy in older persons (aged ≥ 75 years). Similarly, many studies have shown that the larger the scope of an operation is, the more complications there are. This study also showed that patients with extrahepatic organ invasion had a greater risk of complications if organ resection was performed simultaneously.

Frailty is an independent predictor of a high incidence of postoperative adverse events (Shinall et al. 2020). Frailty symptoms in elderly patients should be evaluated, and geriatricians should be consulted for further evaluation if necessary (Ko 2019).

Accurate assessment of frailty in elderly people can help individuals identify high-risk groups as early as possible, predict adverse health outcomes, and provide a reference for further assessment, treatment, and nursing measures for elderly people with different degrees of frailty. In addition, accurate assessment of frailty in perioperative elderly patients can guide doctors in controlling the safety of perioperative procedures. Frailty is associated with poor surgical outcomes and poor prognosis (McIsaac et al. 2017). The risks of surgery and perioperative complications are increased in older people with frailty. Beggs et al. (Beggs et al. 2015) analyzed 19 studies on frailty and perioperative outcomes and found that although the evaluation criteria and types of surgery were different, frailty was associated with perioperative adverse outcomes to some extent. Frail patients have higher mortality, morbidity, and complications; longer hospital stays; and slower recoveries after discharge than nonfrail patients (Makary et al. 2010).

Comorbidities and fatigue are the main conditions used to assess frailty (Church et al. 2020; Thompson et al. 2020). Many patients who require surgery often have one or more other medical conditions, termed comorbidities (Couri and Pillai 2019). Comorbidities are common in elderly individuals and can affect disease manifestation and severity, sometimes even impacting management (Scichilone 2017). Old age, therefore, is associated with a number of age-associated risks and remains the most common predisposing factor for poor postoperative outcomes (Olotu 2021). With the development of traditional surgery, a large number of high-risk surgery patients with single/multiple-organ dysfunction have undergone surgery, and the number of surgical patients with atherosclerosis, diabetes, chronic obstructive pulmonary disease, and other internal diseases has increased rapidly. Fatigue is also significantly related to postoperative adverse events. This study also showed that ≥ 5 kinds of comorbid diseases and fatigue were independent risk factors for postoperative complications in older persons (aged ≥ 75 years).

One study used the abnormal skeletal muscle mass index, type of surgery, and preoperative serum albumin concentration to develop a risk-scoring system for liver surgery in older persons (Tomita et al. 2021). When the risk score of this scoring system was ≤ 1, the postoperative complication rate was 0.0%; when the risk score was ≥ 4, the postoperative complication rate was 57.1%, and the AUC was 0.810. However, at present, abnormal skeletal muscle mass indices are not routinely detected by this evaluation method in clinical practice, which makes this evaluation system unsuitable for widespread use.

The items of RASHA established in this study are easy to obtain clinically, and RASHA has not only a score (RASHA scale) but also a risk probability (RASHA formula), which makes the evaluation results more intuitive. In addition, our research focused on the conditions of older persons, including fatigue, comorbidities, and other factors related to frailty. Therefore, RASHA is more effective at assessing surgical risk in older patients.

Accurate assessment of frailty in older patients during surgery can guide doctors in controlling the safety of surgery. Because short and simple instruments are most feasible in clinical practice, several quick screening tools have been developed and validated. However, these scales have the disadvantages of complicated evaluation processes and difficult data acquisition, which limit their clinical application. For example, the FRAIL scale consists of five items: fatigue, resistance, aerobic, illness, and loss of weight. However, obtaining a specific weight loss and walking distance (resistant or aerobic) is difficult, which limits the application of the FRAIL scale. Therefore, this study used fatigue and comorbidities to reflect the state of frailty. However, if a patient’s frailty can be assessed with a widely recognized frailty assessment tool, the patient's frailty state can be better assessed.

## Conclusion

In this study, a new risk assessment system for hepatectomy in adults older than 75 years (RASHA) was established (including two parts: the RASHA scale and the RASHA formula). As a novel and simplified assessment system, RASHA can be used to predict the postoperative complications of hepatectomy effectively in adults older than 75 years through preoperative factors.

## Data Availability

No datasets were generated or analysed during the current study.
